# Comprehensive review on the application of omics analysis coupled with Chemometrics in gelatin authentication of food and pharmaceutical products

**DOI:** 10.1016/j.fochx.2024.101710

**Published:** 2024-08-03

**Authors:** Putri Widyanti Harlina, Vevi Maritha, Fang Geng, Asad Nawaz, Tri Yuliana, Edy Subroto, Havilah Jemima Dahlan, Elazmanawati Lembong, Syamsul Huda

**Affiliations:** a Department of Food Industrial Technology, Faculty of Agro-Industrial Technology, Universitas Padjadjaran, 45363 Bandung, Indonesia; b Padjadjaran Halal Center, Universitas Padjadjaran, 45363 Bandung, Indonesia; c Pharmacy Study Program, Faculty of Health and Science, Universitas PGRI, Madiun, Indonesia; d Meat Processing Key Laboratory of Sichuan Province, School of Food and Biological Engineering, Chengdu University, Chengdu, 610106, China; e Hunan Engineering Technology Research Center for Comprehensive Development and Utilization of Biomass Resources, College of Chemistry and Bioengineering, Hunan University of Science and Engineering, 425199 Yongzhou, China

**Keywords:** Gelatin, Authentication, Omics, Chemometrics, Food and pharmaceutical

## Abstract

Gelatin is a protein molecule that can be hydrolyzed from collagen, animal bones, skin and it easily soluble in water. Source animals for gelatin ingredients must be evaluated, as well as their halal status. The omics method towards gelatin authentication in food and pharmaceutical products has several advantages, including high sensitivity and reliable data. Omics investigation employs the process of breaking down substances into small particles, hence enhancing the ability to detect a greater number of compounds. Omics study has the capability to identify substances at the subclass level, which makes it highly suitable for gelatin authentication. Gelatin lipids, metabolites, proteins, and volatile chemicals can be utilized as references to authenticate gelatin. In adopting gelatin authentication, lipidomics, metabolomics, proteomics, and volatilomics must be combined with chemometrics for data interpretation. Chemometrics can convert omics analysis data into easily viewable data. Chemometric approaches capable of presenting omics analysis data for gelatin authentication include PCA, HCA, PLS-DA, PLSR, SIMCA, and FACS. Visually chemometrically explain the differences in gelatin from different animal sources. The combination of omics analysis and chemometrics is a very promising technology for gelatin authentication in food and pharmaceutical products.

## Highlights

• Omics analysis is a reliable method for verifying gelatin authenticity.
• Chemometrics like PCA, HCA, PLS-DA, PLSR, SIMCA, and FACS simplify complex data to distinguish gelatin sources.
• These techniques promise to revolutionize quality control and regulation in the food and pharmaceutical sectors.
• The fusion of omics analysis and chemometrics presents a promising approach for addressing gelatin authenticity concerns.

## 1 Introduction

Gelatin is a protein compound that is easily soluble in water (Uddin et al., 2021) and hydrolyzed from collagen, animal bones, and skin. Pigs, cows, goats, fishes, and other types of animals used for gelatin extract (Alipal et al., 2021; Karayannakidis & Zotos, 2016). Gelatin is used in food and pharmaceutical products (Elgadir, Mirghani, & Aishah, 2013; Nur Hanani et al., 2014). In the food industry, gelatin is used as a film for edible food packaging. This provides the advantage of reducing packaging waste in food (Kumar et al., 2018). Gelatin is utilized in cosmetic preparations and as a component in capsule casings in pharmaceutical products (Gullapalli & Mazzitelli, 2017). Gelatin is now used extensively in both industries. The animal resources for gelatin ingredients need to be considered, including their halal status (Shabani et al., 2015).

Halal gelatin in food and pharmaceutical products is currently a concern in many countries, one of which is in Indonesia, where the majority of the population is Muslim (Nirwandar, 2020). Gelatin that comes from non-halal animals would make non-halal gelatin (Nikzad et al., 2017). Non-halal gelatin in food and pharmaceutical products would make these two kinds of products non-halal and unacceptable to consumers (Yusof & Shah, 2014). Therefore, it is necessary to have an analytical method that is able to identify the source of gelatin in food and pharmaceutical products. An analytical method that has good sensitivity in identifying the origin of gelatin is omics analysis, such as lipidomics (Hirata et al., 2021), metabolomics (Harlina et al., 2024, Le et al., 2023), proteomics (Jannat et al., 2018), and volatilomics (Maritha et al., 2023).

Omics analysis is an analytical method based on cellular networks and communication in organisms (Palla et al., 2022). Authentication of gelatin in food and pharmaceutical products using the omics approach has advantages including good sensitivity and accurate data. Lipidomics is a part of omics analysis which is able to see the lipid composition in gelatin. Through a lipidomics approach, the source of gelatin can be known whether it comes from halal or non-halal animals (Blondelle et al., 2017). Another type of omics analysis is metabolomics. Metabolomics is able to see metabolites in gelatin sources, so that which animals are used as ingredients for making gelatin can be known (Le & Yang, 2022). A component of omics analysis called proteomics was able to separate the protein in gelatin from other components of the mixture. In the meantime, volatilomics would separate gelatin through the volatile substances (Amalia et al., 2022). Because the volatile molecules in each species vary, gelatin authentication is compatible with volatilomics. Omics analysis in implementing gelatin authentication requires a combination with chemometrics for data interpretation. Chemometrics is a method to present omics analysis data in a more understandable way (Maritha et al., 2022).

Chemometrics is an analytical tool that may effectively illustrate omics data (Pollo et al., 2021). Chemometrics is able to present omics analysis data into clearly visualized data (Calderon, 2017). Various types of chemometrics, both supervised and unsupervised to authenticate data from omics analyses, both targeted and untargeted. As a result, this strategy would be significantly more capable of explaining the authenticity of gelatin based on omics analysis data (Tortorella et al., 2020).

According to the aforementioned explanation, omics analysis combined with chemometrics is a very promising technique for gelatin authentication in food and pharmaceutical products. This method of verification has the potential to be improved in order to certify the gelatin contained in those products.

## 2 Halal Gelatin in Food and Pharmaceutical Products

The use of gelatin is expanding right now, particularly in the food, pharmaceutical, and cosmetic industries (Ahmed et al., 2020). Gelatin is obtained from the skeletal remains of several animals. Gelatin can be produced from the bones and skins of many animals such as fish, cows, pigs, and goats. The diverse sources of gelatine from various animal species result in variations in its qualities and characteristics. For instance, the composition of metabolites in porcine gelatin may differ from that of bovine and fish gelatin. The varying attributes of gelatine derived from different sources result in distinct formulations for food and cosmetic products (Pradini et al., 2018). Gelatin that comes from various types of animals in food and pharmaceutical products becomes a special problem, especially for Muslims. Gelatin sourced from non-halal animals becomes non-halal gelatin (Abdullah Amqizal et al., 2017). This makes the development of gelatin from halal animals continues to be developed, one of which is from bones and skins of goats and fish (Nurilmala et al., 2022; Sudjadi Wardani, Sepminarti, & Rohman, 2016).

Goats and fish become potential animals as sources of halal gelatin. Gelatin derived from goat skin for gel has properties similar to gelatin from cows (Mad-Ali, Benjakul, Prodpran and Maqsood, 2017). Gelatin derived from goats also has high gel strength properties (Mad-Ali et al., 2016). Another characteristic of goat gelatin is that it has a fine and regular structure. Therefore, goat gelatin as a potential substitute for commercial gelatin (Mad-Ali, Benjakul, Prodpran and Maqsood, 2017). Apart from goats, fish also have the potential to produce halal gelatin. Fish gelatin is obtained from the hydrolysis of fish collagen and contains abundant amino acids for nutritional use as a food product (Lv et al., 2019). The characteristics of fish gelatin show that it is stable against heat (Binsi et al., 2017). Fish gelatin also has properties of good films in food and pharmaceutical products (Tongnuanchan et al., 2013). Fish gelatin is capable of making good films and chitosan as safe food packaging materials (Li et al., 2021). Fish and goat gelatin is good as halal gelatin because it has heat-stable properties and structure and has the ability to become an emulsifier for food and pharmaceutical products.

## 3 Omics Analysis for Authentification of Halal Gelatine in Food Products

### 3.1 Lipidomic Analysis

Lipidomic analysis is an analytical method based on the composition of lipids and sub lipids. Sub lipids is the grouping of lipids into smaller classes based on their similarity in properties (Sandra & Sandra, 2013). Because gelatin is made from animal bones or skin, it contains fatty molecules (Yang et al., 2019). The lipid profile derived from the gelatin of each animal would be different because lipids are unique compounds that are owned by living things (Aksun Tümerkan et al., 2019). The lipid profile such as palmitoleic acid, oleic acid, and linoleic acid in each of these animal gelatins as a reference for gelatin authentication (Garcia Londoño et al., 2022). This method can be applied for authentication of gelatin in food products (Creydt & Fischer, 2022; Dirong et al., 2021). Based on its lipid profile, lipidomics used to selectively discriminate gelatin in food items. This lipid will distinguish the source of gelatin, whether it comes from halal or non-halal animals. Lee and Chin (2016) explored how gelatin influences sausage texture based on fat quantity.

Lipidomics is able to analyze lipids in samples, including gelatin (Holčapek et al., 2018). Fatty acyls, glycerolipids, glycerophospholipids, and sphingolipids are lipid groups that have different profiles in each animal which is used to differentiate gelatin sources. These lipid groups will also alter the profile of gelatin (Zhang et al., 2020). The presence of profiles in various lipid classes in gelatin can be utilized as a reference for determining the authenticity of gelatin in meals. Because gelatin can come from a variety of animals, lipidomics analysis has the ability to authenticate gelatin in food products (Mortas et al., 2022).

### 3.2 Metabolomic Analysis

**Fig. 1:**
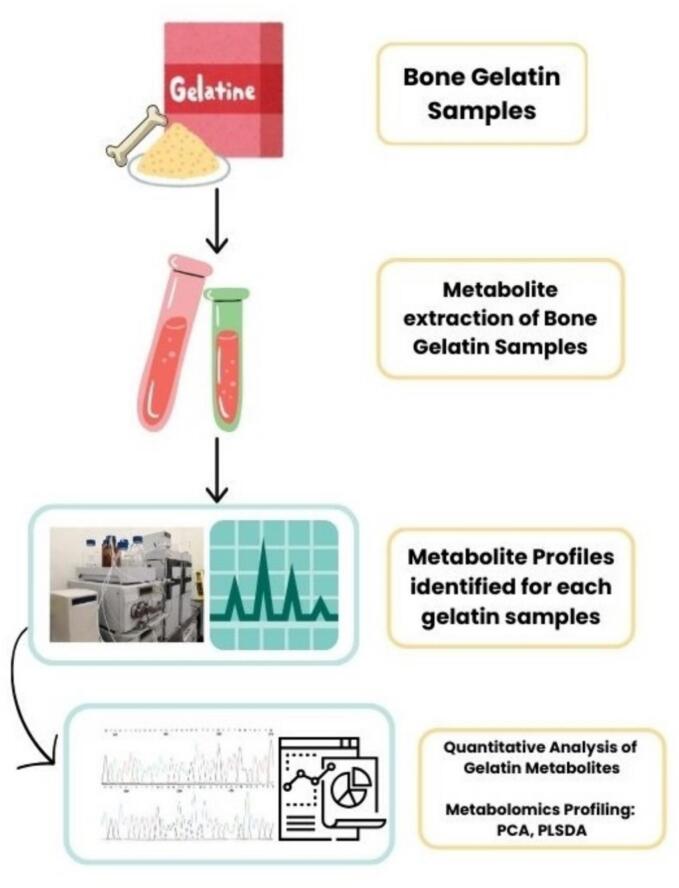
The Workflow of Metabolomics Analysis from Gelatin Samples.

Metabolomic analysis is an analytical method based on the metabolites present in the sample. Gelatin is a metabolite-containing substance (Utpott et al., 2022). Gelatin analysis using metabolomics can offer data on gelatin metabolites (Li et al., 2018). According to Zhao et al. (2019), fish gelatin metabolites change during storage. NMR-based metabolomic research revealed that choline and trimethylamine oxide are metabolites that alter during storage. As a result, metabolomic analysis used to verify the presence of gelatin in food products. The LC-MS (Liquid Chromatography- Mass Spectrometry) method, which incorporates metabolomic analysis in gelatin, is a technology that offers the fingerprint of metabolites such as cretaine, isolusin, and DL-carnitine in biological samples. A combination of chromatographic and mass spectrometry techniques could make sample preparation, data capture, and subsequent processing easier (Moosmang et al., 2019). This method is frequently used to identify and characterize tiny metabolite compounds in gelatin products with good separation. Fig. 1 depicts the workflow of metabolomics analysis from gelatin samples.

### 3.3 Proteomic Analysis

Proteomic analysis is a method of describing the protein profile in a sample. One of the components found in gelatin is protein (Djagny, Wang, & Xu, 2010). Because each animal's protein profile varies, proteomic can be utilized to distinguish distinct types of gelatins. According to Uversky et al. (2021), proteomics can assess gelatin in royal jelly. Zhu et al. (2023), investigated the utilization of 11 peptide biomarkers for verification of pig, bovine, and donkey gelatins using the UPLC-HRMS (Ultra Performance Liquid Chromatography–High-Resolution Mass Spectrometry) equipment. This approach has been used successfully to detect adulteration of pig-derived components in gelatin and to quantify pork gelatin in sweets and dairy products. Collagen alpha-1(I) chain and Collagen alpha-1(II) chain is a peptide that differentiates between porcine, bovine and goat gelatin. Proteomics can be utilized to validate the halal status of gelatin in food products (Zhu et al., 2023).

LC-QTOF-MS (Liquid Chromatography Quadrupole Time of Flight Mass Spectrometry) is a potent instrument for identifying protein peptides that used to determine the type of animal samples (Zamaratskaia & Li, 2017). In the proteomic analysis method, protein extraction starts before MS (Mass Spectrometry) or Time-of-flight analysis. The most common method for identifying proteins or peptides in proteomics is mass spectrometry. This approach has several uses, including animal science research; however it is limited by protein biochemical heterogeneity and the difficulty to detect low protein levels. The instrument is able to detect hundreds of peptides with high accuracy.

### 3.4 Volatilomic Analysis

Volatilomics is a method of analyzing volatile compounds in samples. The GC–MS (Gas Chromatography - Mass Spectrometry) is a popular device for volatilomic analysis. Volatile compounds can be a determinant of gelatin authentification (Qi et al., 2020). As a result, based on the volatile compounds found in gelatin, volatilomic analysis can be utilized to authenticate gelatin (Nurani et al., 2022). According to Mazzucotelli et al. (2022), it may be possible to identify food products using volatile molecules that have been analyzed using PTR-MS (Proton Transfer Reaction Mass Spectrometry). Volatile compounds, such as alcohol derivatives to verify the halal status of gelatin in food products. Alcohol derivatives are non-halal compounds, so authentification of these compounds determines the halalness of the product. Using the volatilomic technique based on the composition of volatile compounds, gelatin from pig, beef, or fish can be identified (Nurani et al., 2022).

The profiles of volatile compounds in each animal species vary. Volatile compounds observed in animals include xylene, benzene, and formaldehyde (Davidson et al., 2021). Volatile compounds found in animals will likewise be found in gelatin derived from animals. The volatile component profiles of pigs, cows, and fish can vary (Ali et al., 2017). The difference in volatile compounds in each animal could also give a profile to the gelatin produced from that animal. Gelatin from fish used in food products would have a different profile than gelatin from other animals (Awuchi et al., 2022). As a result, volatilomics used to authenticate gelatin in food products.

## 4 Omics Analysis for Authentication of Gelatin in Pharmaceutical Products

### 4.1 Lipidomic Analysis

Lipidomic analysis can be utilized to authenticate gelatin not only in food products, but also in medicinal and cosmetic products (Guazzotti et al., 2023). Highly distinct lipids can assist in determining the source of gelatin in pharmaceutical products. Lipidomics can identify between different types of fish bones, and because fish bones are used to make gelatin, this method of analysis can distinguish between different types of gelatin. Several lipids can be utilized as references for authenticating fish bones. Lipids used for fish bone authentification are tiglycerides (TGs), phosphatidylcholines (PCs), phosphatidylethanolamines (PEs), and ceramides (Cers) (Yan et al., 2022). One of the promising techniques is lipidomic authentication for gelatin in pharmaceutical products (Ali et al., 2017).

Lipidomics is an approach used in the pharmaceutical sector that can ensure the gelatin quality (Mahrous, Chen, Zhao, & Farag, 2023). Gelatin used in capsule casings comes from a variety of sources, depending on its halal status. The use of porcine gelatin capsule casings is certain to render this product non-halal. Lipidomics can tell the difference between capsule casings made from porcine gelatin and those that aren't (Bosch-Queralt et al., 2021). Lipidomics can even detect the origin of gelatin in capsule casings when gelatin is mixed. This approach is highly sensitive for identifying gelatin in medicinal products (Li et al., 2022).

### 4.2 Metabolomic Analysis

Gelatin is frequently utilized in pharmaceutical products. Gelatin is used to make semisolid products, such as cosmetics (Sultana, Ali, & Ahamad, 2018). Considering gelatin utilized in pharmaceutical products is derived from animals, an analytical method, such as metabolomics (Abedinia et al., 2020), is required to authenticate gelatin in pharmaceutical products. Since peptides found in gelatin in pharmaceutical products can be identified using metabolomics, gelatin authentication using this method could be achieved (Liu, Nikoo, Boran, Zhou, & Regenstein, 2015). Differences in peptides between animals to authenticate gelatin in medicinal compositions using the metabolomic method. In mass spectrometry, chromatography is used to separate metabolites from samples. UPLC (Ultra Performance Liquid Chromatography) is typically used to separate metabolites due to its excellent separation capabilities. The TOF-MS (time-of-flight mass spectrometry) apparatus could be coupled to the UPLC apparatus, making it simpler to acquire the research data (Harlina et al., 2022).

The pharmaceutical industry's choice of gelatin source is impacted by a number of factors, including its wide availability, low cost, and favorable characteristics (Alfaro et al., 2015). These factors account for the widespread usage of pig gelatin in pharmaceutical products (Elgadir, Mirghani, & Aishah, 2013). However, this brings up the fact that Muslims are prohibited from consuming pork. Gelatin's halalness can be verified using the metabolomics technique. Pig gelatin contains different amino acids than other animal gelatins, including that found in cow. In order to differentiate between halal and non-halal gelatin in pharmaceutical products, this difference in profile can be employed as a guide (Castro-Muñoz et al., 2021).

### 4.3 Proteomic Analysis

Gelatin is a protein-based substance derived from bone hydrolysis, primarily of animal origin. The origin of gelatin used in pharmaceutical products, as well as its halal status, is of importance in particular (Hameed et al., 2018). As a result, it is critical to verify the halal status of gelatin in pharmaceutical goods. Ward et al. (2018), investigated whether UPLC-MS/MS (Ultra Performance Liquid Chromatography-Tandem Mass Spectrometry) proteome analysis could detect pig, beef, chicken, and fish gelatins used in pharmaceutical products. According to Uechi et al. (2014), proteomics can identify peptides in capsules. Based on the information provided above, gelatin authentication can is accomplished using the proteomic technique, which is an analysis, based on protein components in the samples.

A product made from animal bones called gelatin has high protein content. The protein profile in gelatin produced from diverse types of animals is likely to differ since the amino acid composition of each animal may vary (Chandra & Shamasundar, 2015). Gelatin authenticity may be determined by variations in the protein content. Bovine, fish, and hog gelatin all contain variable amounts of protein (Karim & Bhat, 2009). The authenticity of the gelatin, including whether it is halal, depends on this discrepancy. The identification of gelatin in pharmaceutical products can be determined by the various protein profiles of each animal gelatin (Sultana, Hossain, et al., 2018).

### 4.4 Volatilomic Analysis

Volatilomics is one of the omics analyses used to validate gelatin in pharmaceutical products. Volatile compounds contained in gelatin would be the determining compounds for gelatin authentication in pharmaceutical products (Elgadir & Mariod, 2022). Gelatin is used as a drug delivery agent in pharmaceutical preparations, then these preparations will enter the blood vessels, if the gelatin used comes from non-halal animals, then these drug preparations will become non-halal (Foox & Zilberman, 2015). Rawdkuen et al. (2013), discovered that volatilomics utilizing GC (Gas Chromatography) could differentiate gelatin from several types of fish. This demonstrates that volatilomics for authentication of gelatin in pharmaceutical products.

**Fig. 2:**
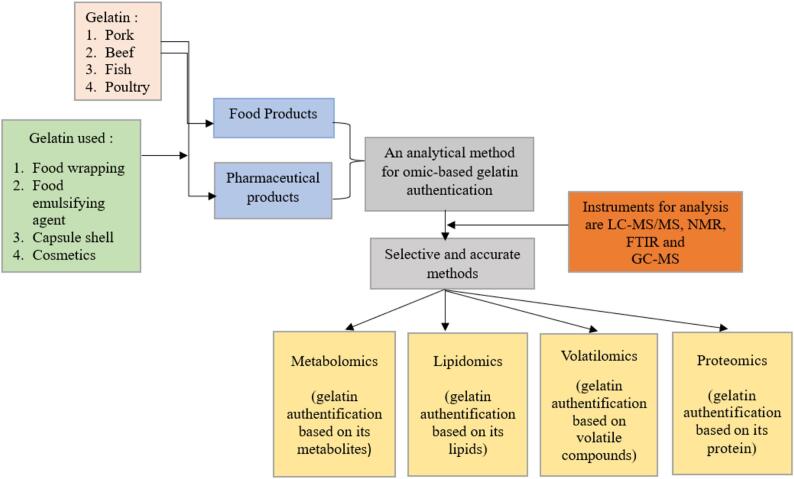
Authentication of Gelatin in Food and Pharmaceutical Products using Omics Approach.

Volatilomics offers good selectivity when used for gelatin authentication in pharmaceutical products (Mohamad et al., 2022). Each animal species' distinctive chemical compounds as the identifier for authentication. Since gelatin is a substance made from animal bones, the volatile compounds can differ depending on the animal (Gómez-Guillén et al., 2009). The distinguishing element for authentication lies in this particular profile of volatile compounds in gelatin. A summary of omics analyses for halal authentication of gelatin in food and pharmaceutical products is presented in Table 1 and Fig. 2.

**Table 1:** Omics Analysis of Gelatin in Food and Pharmaceutical Products.

**No**	**Author**	**Title**	**Omics Analysis**	**Importance Results**	**Ref**
1	Ma, T, et al.	EGCG-gelatin biofilm improved the protein degradation, flavor, and micromolecule metabolites of tilapia fillets during chilled storage	Metabolomics	Metabolomics with UHPLC-Q-TOF/MS analysis indicated a distinct separation between the CON and treatment groups at the end of storage	(Ma et al., 2022)
2	Chandra and Shamasundar	Texture Profile Analysis and Functional Properties of Gelatin from the Skin of Three Species of Fresh Water Fish	Metabolomics	Metabolomics was able to see the metabolite profile of gelatin	(Chandra & Shamasundar, 2015)
3	Pereira et al.	Anthocyanin-sensitized gelatin-ZnO nanocomposite based film for meat quality assessment	Volatilomics	Spearman correlation between the response and the total volatile basic nitrogen released during meat spoilage for both storage conditions.	(F.M. Pereira, De Sousa Picciani, Calado and Tonon, 2022)
4	Chawla, S. et al.	The effect of silk–gelatin bioink and TGF-β3 on mesenchymal stromal cells in 3D bioprinted chondrogenic constructs: A proteomic study	Proteomics	hMSCs-laden bioprinted constructs contained a large percentage of collagen type II and Filamin-B, typical to the native articular cartilage.	(Chawla et al., 2021)
5	Yilmas, M. et al.	A novel method to differentiate bovine and porcine gelatins in food products: NanoUPLC-ESI-Q-TOF-MSE based data independent acquisition technique to detect marker peptides in gelatin	Proteomics	The marker peptides specific for porcine and bovine could be successfully detected in the gelatin added to the dairy products analyzed, revealing that the detection of marker peptides in the digested gelatin samples using nanoUPLC-ESI-q-TOF-MS^E^ could be an effective method to differentiate porcine and bovine gelatins in the dairy products	(Yilmaz et al., 2013)
6	Wang et al.	Comparative lipidomics analysis of human, bovine, and caprine milk by UHPLC-Q-TOF-MS	Lipidomics	A total of 13 lipid classes (including TG, DG, SM, PC, Cer, HexCer, Hex_2_Cer, PE, PG, PS, PI, PA and CL) were successfully analyzed.	(Wang et al., 2020)

Table 1 describes the different types of omics analysis capable of authenticating gelatin. Gelatin from three types of freshwater fish skin has a different metabolite profile. Metabolites are the result of gene expression derived from the interaction between the genomic system and the environment, so each species will have different metabolites including metabolites from gelatin. This makes metabolomics a method that to authenticate gelatin. Gelatin is a compound composed of proteins, so proteomics is an appropriate method for gelatin authentication. Proteins are macromolecular compounds found in organisms, where each organism will have a different protein profile. Proteins in cattle and pigs are clearly different, so bovine and porcine gelatins, which are composed of the majority of proteins, can be distinguished by proteomic methods. In addition to metabolites and proteins, lipids are also compounds that are unique to each living thing. Animal-sourced gelatin will have lipids in it. Lipids in gelatin from different types of animals will show different types, so lipidomics will be able to authenticate gelatin from various animal species.

## 5 Application of Chemometrics for Authenticating the Halal Status of Gelatin in Food Products

Combining chemistry and statistics, chemometrics simplifies data presentation (Kanginejad & Mani-Varnosfaderani, 2018). Increasingly, chemometrics is used to manage chemical-related data due to its efficacy in data-solving applications such as metabolomic and lipidomic analyses (Feizi et al., 2021; Maritha et al., 2022; Navarro-Reig et al., 2018). It enables the control of the number of variables involved in the analysis (Karabagias, 2020) and provides precise and statistically significant results in a brief period of time, in addition to having a high sensitivity and stability (Wu et al., 2021). Chemometrics is a frequently used method for halal authentication, including in food products (Harlina et al., 2022). Chemometrics can effectively offer data for the authenticity of food products such as gelatin. Several chemometric methods have been used to ascertain the origin of gelatin (Garcia-Vaquero & Mirzapour-Kouhdasht, 2023). According to Azira et al. (2012), PCA (Principal Component Analysis) was able to differentiate pork and beef gelatins based on peptides in processed food. PLS-DA (Partial Least-Squares Discriminant Analysis) is also able to separate beef and pork gelatins in food products using Raman spectroscopy (Forooghi et al., 2023). HCA (Hierarchical Cluster Analysis) is another chemometric analysis approach to authenticate gelatin in food products. HCA categorizes gelatin derived from various fruit and fish extracts for use in gummy jelly products (Renaldi et al., 2022). In addition, FACS (Fuzzy Auto Catalytic Set) is another chemometric approach used to certify gelatin in food products. This approach can regulate the gelatin quality in fish during preservation (Moula Ali et al., 2020).

PCA is a technique used to decrease the number of dimensions in specific datasets. Enhances comprehensibility while minimizing information loss. It accomplishes this by generating new covariates that are independent of one another. Identifying the additional variables, also known as the principal components, will simplify the challenge of solving for eigenvalues and eigenvectors. PCA is considered an adaptable data analysis technique because to its ability to produce variables that may adapt to various types and structures of data (Mohammed et al., 2021). The PLS-DA algorithm is highly adaptable and can be utilized for both predictive and descriptive modeling, as well as for discriminative variable selection. PLS-DA has proven to be highly effective in modeling datasets with a large number of variables for various applications. These include verifying the authenticity of products in food analysis, classifying diseases in medical diagnosis, and analyzing evidence in forensic research (Lee et al., 2018). Agglomerative hierarchical clustering is distinct from partition-based clustering in that it constructs a binary merge tree, beginning with individual data components as leaves and culminating in the root node that encompasses the entire dataset. A dendrogram is the graphical representation of a tree that displays the nodes on a plane. In order to carry out a hierarchical clustering technique, it is necessary to select a linkage function, such as single linkage, average linkage, complete linkage, Ward linkage, etc. This function determines the distance between any two subsets, based on the underlying distance between elements (Cohen-addadVincent et al., 2019).

## 6 Application of Chemometrics for Authenticating the Halal Status of Gelatin in Pharmaceuticals Products

**Fig. 3:**
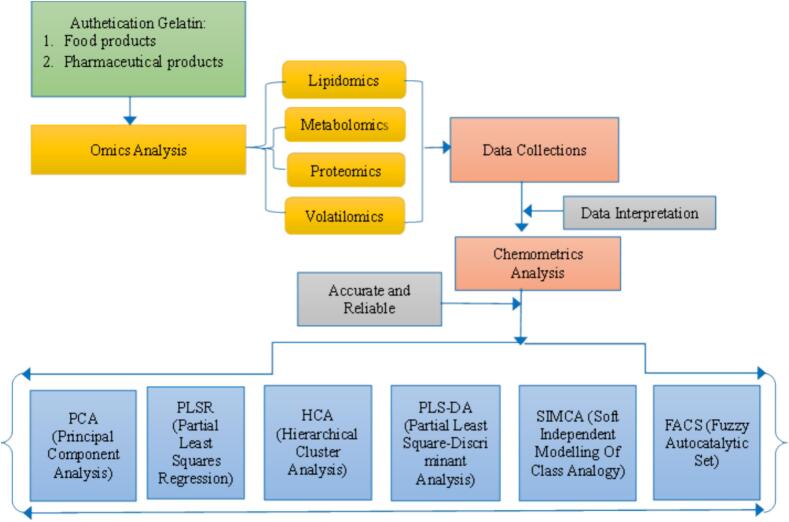
Chemometric Analysis for Authentication of Gelatin in Food and Pharmaceutical Products.

Besides being used for authentication of gelatin in food products, chemometric analysis is also used in pharmaceutical products (Esteki et al., 2018; Xu et al., 2020). Widyaninggar et al. (2012) demonstrated how to discriminate beef and pork gelatins in capsule shells based on protein profile using PCA based on the score plot of the first principal component (PC1) and the second principal component (PC2). PLS-DA as a chemometric method for authentication of gelatin in pharmaceutical products. PLS-DA has the ability to evaluate highly collinear and noisy data (Gromski et al., 2015), particularly when the number of variables involved exceeds the amount of samples. PLS-DA can classify pig gelatin from diverse manufacturers in the authentication of gelatin for hard capsules (Duconseille et al., 2017). PLSR (Partial Least Squares Regression) is also used in the authentication of gelatin in pharmaceutical food products. Based on the protein components included in gelatin, this approach may determine the type of gelatin used in microencapsulation (Vongsvivut et al., 2014). Furthermore, SIMCA (Soft Independent Modeling by Class Analogy) is also capable of classifying gelatin in capsule casings based on its source (Debnath et al., 2022). In omic research, the combination of more than one chemometric approach is often used to provide proper analysis and data interpretation (Roberts et al., 2008). Authentication of gelatin using chemometric methods is presented in Fig. 3.

Fig. 3 depicts numerous chemometric approaches for identifying gelatin in food and pharmaceutical products. Each chemoemtric method has a distinct advantage in terms of gelatin authentication. PCA offers the advantage of reducing data so that differences between samples are more visible (Maione & Barbosa, 2018), yet PLS-DA can predict the deciding components for gelatin authentication such as lipids, metabolites, proteins, and volatile compounds (Jiménez-Carvelo et al., 2021). HCA also offers the advantage of grouping samples, such that samples with similar properties are grouped to identify the origin of gelatin (Granato et al., 2018). SIMCA has the advantage of being able to focus on certain categories, therefore this method can determine how much of a sample belongs to a specific class, and to classify gelatin varieties (Di Donato et al., 2023). Chemometric analyses used for authentication of gelatin in food and pharmaceutical products are presented in Table 2.

**Table 2:** Chemometric Analyses of Gelatin in Food and Pharmaceutical Products.

**No**	**Author**	**Title**	**Chemometric Analysis**	**Importance Results**	**Ref**
1	Hassan, N. et al.	Identification of bovine, porcine, and fish gelatin signatures using chemometrics fuzzy graph method	PCA, LDA, and c-FACS	The c-FACS method was rigor and faster than PCA and LDA in differentiating the gelatin sources	(Hassan et al., 2021)
2	Acevedo et al.	Assessment of gelatin–chitosan interactions in films by a chemometric approach	PLSR	PLSR showed that thermal properties of gelatin–chitosan films were affected by the presence of amino acids Cysteine, Tyrosine, Valine, Arginine, Hydroxyproline, and Glycine	(Acevedo et al., 2015)
3	Jannat et al.	Distinguishing tissue origin of bovine gelatin in processed products using LC/MS technique in combination with chemometric tools	PCA and PLS-DA	PCA and PLS-DA were used to classify samples as either skin or bone gelatins	(Jannat et al., 2020)
4	Cebi et al.	A rapid ATR-FTIR spectroscopic method for classification of gelatin gummy candies in relation to the gelatin source	HCA, PCA, and PLS-DA	The spectral region between 1734 and 1528 cm^−1^ was selected for chemometric analysis	(Cebi et al., 2019)
5	Raraswati et al.	Differentiation of Bovine and Porcine Gelatins in Soft Candy Based on Amino Acid Profiles and Chemometrics	PCA	Based on PC1 and PC2, porcine and bovine gelatins in soft candy could apparently be distinguished	(Amalia Raraswati et al., 2014)
6	Salamah et al.	Differentiation of Bovine and Porcine Gelatins Using Lc-Ms/Ms. and Chemometrics	PCA	PCA offered a reliable method for differentiation between porcine and bovine gelatins	(Salamah et al., 2019)
7	Mustaqimah et al.	Identification of Gelatin in Dental Materials using the Combination of Attenuated Total Reflection-Fourier Transform Infrared (ATR-FTIR) Spectroscopy and Chemometrics	PCA and SIMCA	Based on the chemometric analysis, one sample (DM-6) had similar profile with porcine gelatin	(Mustaqimah & Roswiem, 2020)
8	Hassan et al.	A Fuzzy Graph Based Chemometric Method for Gelatin Authentication	c-FACS	The results from the c-FACS analysis showed distinct features of each gelatin, particularly porcine gelatin.	(Hassan, Ahmad, Zain, & Awang, 2020)
9	Roswiem, A. and Mustaqimah, D.	The Identification of Gelatin Source in Toothpaste products Using the Combination of Attenuated Total Reflection-Fourier Transform Infrared (ATR-FTIR) Spectroscopy and Chemometrics	PCA and SIMCA	Chemometrics could not provide excellent illustration of the origin of gelatin in the toothpaste products	(Roswiem & Mustaqimah, 2020)
10	Hassan et al.	A Novel Chemometric Method for Halal Authentication of Gelatin in Food Products	FACS	Fuzzy Autocatalytic Set (FACS) to determine the FTIR spectra of bovine, porcine, and fish gelatins	(Hassan, Ahmad, Zain, & Ashaari, 2020)

## 7 Conclusion and Future Perspectives

Omics analysis, which includes lipidomics, metabolomics, proteomics, and volatilomics, is a method with high selectivity for detecting gelatin in food and pharmaceutical products. Metabolites, proteins, and volatile compounds found in gelatin as references for authenticating gelatin. The massive amount of data generated by omics analysis necessitates a mechanism for simplifying and simplifying its presentation. Chemometrics is a method capable of reducing and presenting omics analysis data to authenticate gelatin. PCA, HCA, PLS-DA, PLSR, SIMCA, and FACS are chemometric methods that are capable of presenting omics analysis data for gelatin authentication. The combination of omics analysis with chemometrics is a very promising method for authentication of gelatin in food and pharmaceutical products.

## Funding

This study was funded by Universitas Padjadjaran, Indonesia, under the Internal Funding of Universitas Padjadjaran (Grant Review Article, No. 2291/UN6.3.1/PT.00/2024).

**Institutional Review Board Statement:** Not applicable.

**Informed Consent Statement:** Not applicable.

## CRediT authorship contribution statement

**Putri Widyanti Harlina:** Writing – review & editing, Writing – original draft, Validation, Supervision, Project administration, Investigation, Funding acquisition, Data curation, Conceptualization. **Vevi Maritha:** Writing – original draft, Investigation, Data curation. **Fang Geng:** Writing – review & editing, Supervision. **Asad Nawaz:** Writing – review & editing, Supervision. **Tri Yuliana:** Writing – review & editing, Methodology, Data curation. **Edy Subroto:** Writing – review & editing, Methodology, Data curation. **Havilah Jemima Dahlan:** Writing – review & editing, Methodology, Data curation. **Elazmanawati Lembong:** Visualization, Formal analysis. **Syamsul Huda:** Visualization, Formal analysis.

## Declaration of competing interest

The authors declare that they have no known competing financial interests or personal relationships that could have appeared to influence the work reported in this paper.

## Data Availability

The data presented in this study are available on request from the corresponding author.
